# Structural changes in the retinal layers of patients with Steinert disease

**DOI:** 10.3389/fopht.2026.1959492

**Published:** 2026-10-07

**Authors:** Beatriz Cordón, Beatriz Pardiñas, Mónica Hernández, María Jesús Rodrigo, Elvira Mayordomo, Luis Emilio Pablo, Elena García-Martin

**Affiliations:** 1 Miguel Servet Ophthalmology Research and Innovation Group (GIMSO), Aragon Institute for Health Research (IIS Aragón), University of Zaragoza, Zaragoza, Spain; 2 Department of Ophthalmology, Miguel Servet University Hospital, Zaragoza, Spain; 3 Department of Neurology, Miguel Servet University Hospital, Zaragoza, Spain; 4 Department of Computer Science, University of Zaragoza, Zaragoza, Spain; 5 Aragon Institute of Engineering Research (I3A), University of Zaragoza, Zaragoza, Spain

**Keywords:** ganglion cells, OCT, optical coherence tomography, retinal layers, retinal nerve fiber layer, Steinert disease

## Abstract

**Purpose:**

The purpose of this study was to evaluate the different retinal layers of the posterior pole protocol and peripapillary areas in optical coherence tomography (OCT) to identify significant changes attributable to Steinert disease.

**Methods:**

We analyzed 128 eyes of healthy control subjects and 79 eyes of patients with Steinert disease. We performed the posterior pole and Nsite analytics peripapillary protocols on all subjects and compared thicknesses in the retinal nerve fiber layer (RNFL), ganglion cell layer (GCL), inner plexiform layer (IPL), inner nuclear layer (INL), outer plexiform layer (OPL), outer nuclear layer (ONL), retinal pigment epithelium (RPE), and complete retina (RETINA).

**Results:**

Patients with Steinert disease showed significant alterations in retinal layer thickness compared with age-matched controls. Overall, a generalized thickening of retinal layers was observed; significant thickening of the RNFL was also found in the temporal area of the optic nerve and in the papillomacular bundle, which comprises the retinal ganglion cell axons connecting the macula with the optic nerve.

**Conclusions:**

Steinert disease is associated with retinal structural alterations in retinal layer structure, OCT may provide potential structural markers of retinal involvement in Steinert disease.

## Introduction

Steinert disease, or type 1 myotonic dystrophy (DM1), is caused by an abnormal expansion of the CTG triplet in the untranslated region of the DMPK gene. This disease impacts multiple body systems. It is primarily characterized by progressive muscular weakness and myotonia. In addition to muscular manifestations, patients may experience a variety of ocular symptoms, cardiac issues, and endocrine dysfunction, rendering it a multisystemic disease (1).

It leads to production of dysfunctional proteins that impair cell function. Due to the genetic and epigenetic heterogeneity of the disorder, symptom severity and age of onset vary significantly. Diagnosis is often made through clinical evaluation, followed by genetic testing to confirm the presence of the mutation (2).

From a muscular perspective, DM1 primarily manifests as severe and progressive muscle weakness and atrophy. Myotonia, characterized by muscles’ inability to relax following contraction, is a distinctive symptom of this condition (3). Muscle weakness can impede performance of daily activities, including those requiring prolonged visual effort, potentially contributing to ocular fatigue and increased susceptibility to visual problems (4).

Research findings on ocular manifestations associated with Steinert disease are limited, but reported ocular complications include ptosis, muscular weakness affecting ocular motility, and cataracts (5, 6). Cataracts may be the most relevant condition in this disease, as they are present in nearly all patients affected by this disorder (7). Other features of myotonic dystrophy include slow pupillary response, low intraocular pressure, changes in the retinal pigment epithelium (RPE), degeneration of the retinal nerve fiber layer (RNFL) and alterations in the structure of the macula. These retinal changes may be associated with disease progression and could serve as biomarkers for assessing symptom severity (8).

Regarding the retina, patients with DM1 present significant retinal changes, such as pigmentary degeneration and alterations in retinal layer structure (9). These changes can be evaluated using techniques such as optical coherence tomography (OCT) to assess retinal layer thickness and detect anomalies that may be associated with neurodegenerative diseases (10). There are no conclusive studies to date establishing a direct correlation between retinal findings and the severity of neuromuscular symptoms (e.g. muscle weakness and myotonia) in patients with Steinert disease. However, the presence of retinal alterations in these patients suggests that the multisystemic spectrum of the disease may include the retina, opening the possibility for future studies to investigate whether these changes reflect neuron damage in other regions of the nervous system (11, 12). Unlike previous studies, our study excluded concomitant retinal pathologies, such as epiretinal membranes and macular edema, to more accurately characterize changes in retinal layer thickness attributable to Steinert disease.

The findings of this paper highlight the importance of ophthalmological evaluation of patients with Steinert disease and open up new avenues for research into the pathology, as well as for development of treatments that address its systemic manifestations. Retinal changes may be indicative of the progression of the condition or of central nervous system affectation. These findings not only enrich our understanding of the pathology, but also underscore the need for comprehensive patient management that includes regular ophthalmological evaluation. As research advances, it is hoped that more effective strategies will be developed to treat and care for those suffering from this disease.

## Methods

We assessed 79 eyes from 79 patients with type 1 myotonic dystrophy diagnosed and confirmed by a specialized neurologist (Steinert disease group, STN) and CTG triplets recorded, and 128 eyes from 128 healthy individuals (control group).

The control group included healthy subjects who had undergone both Ppole and Nsite protocols. To increase the reference sample size for the Ppole analysis, additional healthy controls from an existing normative database were included when Ppole recordings were available. As Nsite data were not available for these additional subjects, the number of control eyes differed between protocols (Ppole, n = 128; Nsite, n = 39). All patients with Steinert disease underwent both protocols.

One eye was randomly selected from each participant to ensure the independence of observations. Since both eyes of the same individual are highly correlated due to shared genetic, anatomical, and disease-related factors, including both eyes as independent observations could underestimate variability and overestimate statistical significance. Therefore, the use of a single eye per subject helps avoid these potential biases.

Eyes with abnormal axial length, including short axial length (<22.1 mm) or marked axial elongation (>25.2 mm), were excluded from the study. Eyes with refractive errors ≥5 diopters (D) of spherical equivalent or ≥3 D of astigmatism were also excluded. In addition, a comprehensive ophthalmological examination, including intraocular pressure measurement, was performed to detect ocular abnormalities such as macular or optic nerve damage, moderate-to-severe cataracts, or media opacities. Only subjects with scores of 0 or 1 in the Lens Opacities Classification System III (LOCS III) (13) were included. Structural measurements of the retina were taken using the Spectralis OCT device (Heidelberg Engineering, Germany). The posterior pole protocol (Ppole) and Nsite analytics module (Nsite) were applied to all subjects (**Figure 1**).

**Figure 1:**
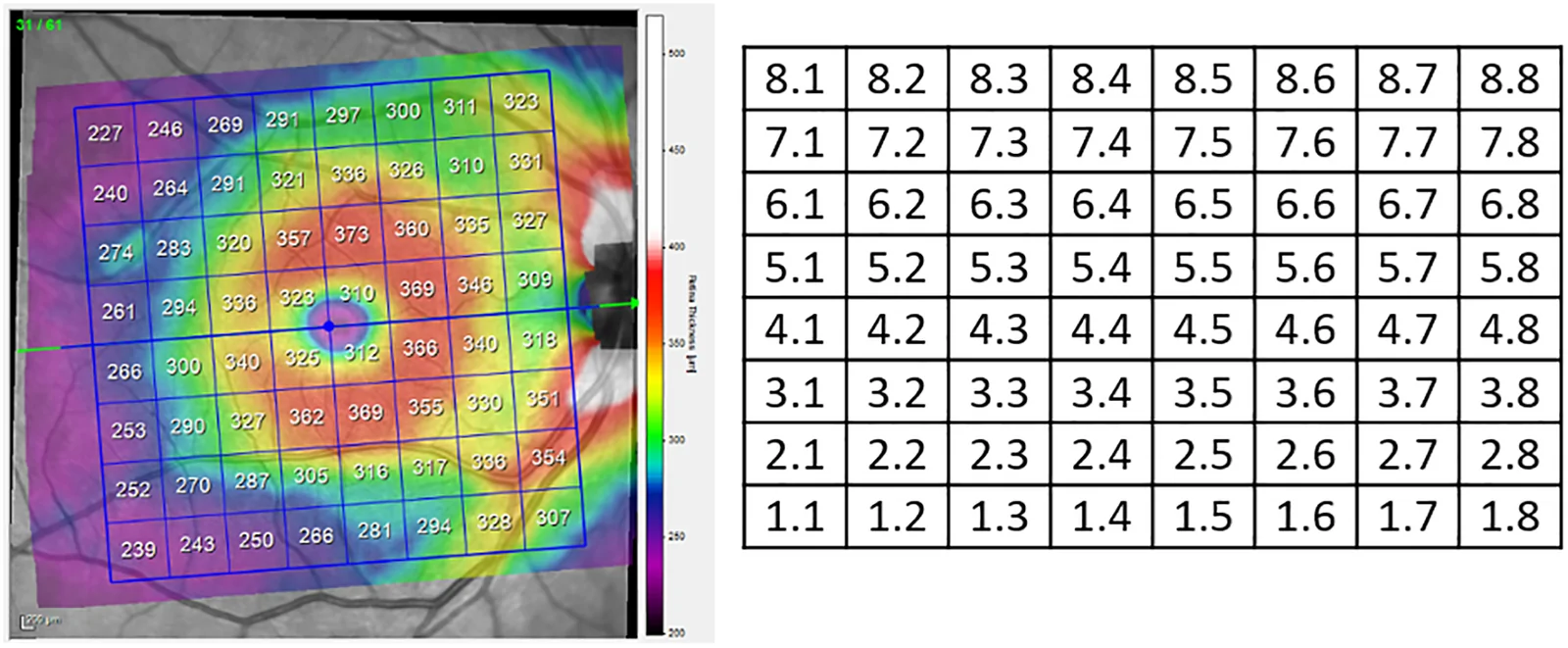
On the left, an en-face image centered on the macula with the posterior pole protocol grid overlay indicating the thickness in each cell of the complete retina layer. On the right, cell identifiers in the posterior pole grid.

- The Ppole protocol allows detailed segmentation of the retinal layers. It incorporates the Anatomic Positioning System (APS), which describes a horizontal line between the fovea and the Bruch’s membrane opening. Based on that reference line, 61 parallel explorations are performed inside a 25° × 30° area. Use of the APS and True Track systems ensures accurate macula positioning based on subject head tilt and eye cyclotorsion. This 25° × 30° area is divided into an 8 × 8 grid which provides global retinal thickness and segments each layer thickness into 64 independent cells. The Spectralis OCT device has an axial (in tissue) resolution of 3.9 μm. The thickness value obtained is the average of each cell (1 × 1 mm). This protocol also provides a scaled color map showing visual changes of 10–15 µm, thus permitting improved detection on the retinal thickness map of small tissue thickness losses. This study evaluated the retinal nerve fiber layer (RNFL), ganglion cell layer (GCL), inner plexiform layer (IPL), inner nuclear layer (INL), outer plexiform layer (OPL), outer nuclear layer (ONL), retinal pigment epithelium (RPE) and complete retina (RETINA). (**Figure 1**).
- The Nsite analytics module provides comprehensive analysis of the peripapillary retinal nervefiber layer. This protocol generates a thickness map showing the mean thicknesses of the 6 sectors (superonasal, nasal, inferonasal, inferotemporal, temporal, and superotemporal). RNFL defects are identified by comparing measurements from each patient against the normative database. This protocol uses fovea-to-disc (FoDi) technology to align subject anatomy and so ensure papillomacular bundle (PMB) measurement accuracy and minimize variability due to patient head orientation. The RNFL-N system centers the viewing window on the temporal region of the scan to facilitate analysis of axonal loss inthe PMB, as observed in patients with neurological diseases. (**Figure 2**).

**Figure 2:**
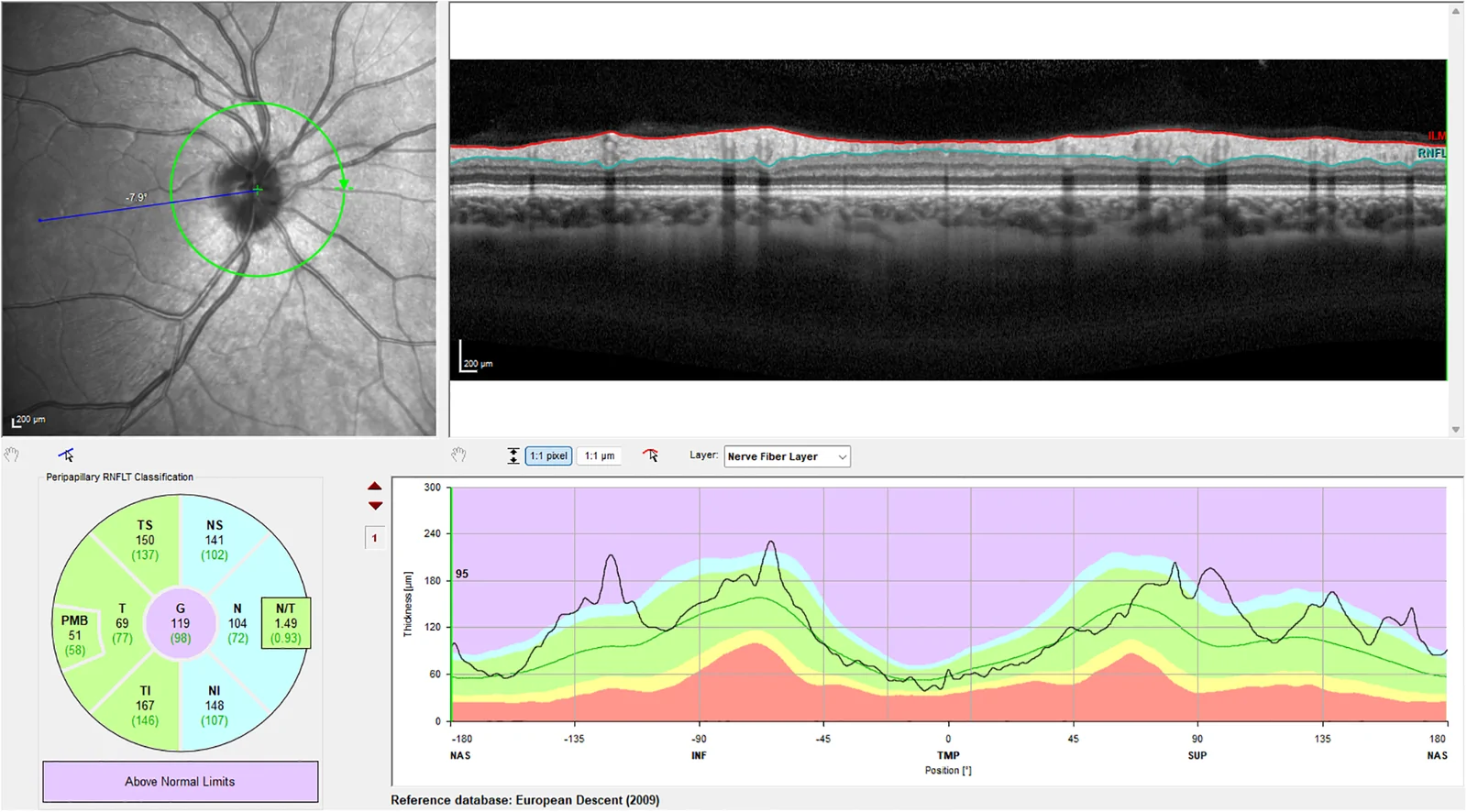
Peripapillary B-scan. Top left, image of the optic nerve captured using optical coherence tomography. Bottom left, thickness grid representing the different areas as per the thickness color diagram based on normalized data. Top right, representation of the B-scan in gray scale. Bottom right, graph showing thicknesses in the different sectors.

No manual correction was applied to the OCT output. An internal fixation target was used because it is reported to have the highest reproducibility. All measurements were obtained by a single operator blind to group classification. The quality of the scans was assessed prior to analysis, and scans with poor image quality or incorrect segmentation were excluded from the analysis. The Spectralis OCT device uses a blue quality bar to indicate signal strength. The quality score ranges from 0 (poor quality) to 40 (excellent quality). Based on previously published studies on the effect of image quality on tissue thickness measurements using similar devices, only images with a quality score > 25 were included in the analysis (14). Consequently, the final sample size was reduced due to exclusion of scans that did not meet these predefined image quality criteria.

Statistics, Since the variables did not follow a normal distribution, the Mann–Whitney U test was performed for each cell across the different retinal layers, as well as for the inner retina, outer retina, and complete retina groupings. P-values were adjusted using the Benjamini-Hochberg false discovery rate (FDR) procedure (q = 0.05).

## Results

The median CTG repeat length in patients with DM1 was 433 repeats (IQR, 148–667; range, 56–1667). In our cohort, patients with Steinert syndrome exhibited a median CTG repeat length greater than 400. This finding should be interpreted in light of the variability in CTG repeat length across different clinical presentations (15).

Both the Ppole and Nsite protocols were performed in the Steinert disease group. In the control group, a subset of healthy subjects underwent both protocols. To increase the sample size, additional healthy controls from our database were included; these subjects had undergone only one of the two protocols.

- OCT-based Ppole analysis was performed on 128 control group eyes [mean age, 42.85 ± 9.21 years; intraocular pressure, 12.24 ± 2.86 mm Hg; gender distribution, 32 males (25%), 96 females (75%)] and on 65 Steinert disease group eyes [mean age, 42.20 ± 13.84 years; intraocular pressure, 11.65 ± 2.14 mm Hg; gender distribution, 28 males (43.08%), 37 females (56.92%)].
- OCT-based Nsite analysis was performed on 39 control group eyes [mean age, 45.02 ± 10.04 years; intraocular pressure, 11.48 ± 1.96 mm Hg; gender distribution, 7 males (17.95%), 32 females (82.05%)] and on 61 Steinert disease group eyes [mean age, 43.27 ± 13.61 years; intraocular pressure, 13.59 ± 2.69 mm Hg; gender distribution, 30 males (49.18%), 31 females (50.82%)].

The means for each of the 64 cells were obtained for both groups and for all retinal layers, and for 8 areas of the peripapillary RNFL. B-scan (**Table 1**).

**Table 1:** Mean retinal layer (Ppole) and peripapillary RNFL (Nsite) thicknesses measurements by optical coherence tomography in the Steinert disease and control groups.

Ppole	Group	RNFL	GCL	IPL	INL	OPL	ONL	Out_Ret	RPE	Inner_Ret	Retina
*1.1*	Steinert	32.13	22.33	18.11	24.95	23.89	47.42	75.53	11.42	168.7	244.28
	Control	29.17	21.59	17.71	23.55	22.27	46.70	75.75	11.90	161.09	236.90
*1.2*	Steinert	37.31	23.64	19.31	26.52	23.97	47.61	75.86	11.73	178.31	254.22
	Control	34.70	23.05	18.67	24.70	22.66	47.02	75.76	11.91	170.73	246.47
*1.3*	Steinert	43.35	24.28	20.18	28.77	24.58	47.82	76.23	11.89	189.00	265.20
	Control	41.27	23.96	19.68	25.69	23.14	47.03	75.64	11.79	180.75	256.38
*1.4*	Steinert	50.60	25.29	21.57	29.43	25.54	47.97	76.66	12.26	200.32	277.00
	Control	48.37	24.91	20.56	26.61	23.79	47.09	76.05	12.14	191.26	267.28
*1.5*	Steinert	60.15	25.35	21.45	28.88	26.11	47.74	76.83	12.37	209.82	286.66
	Control	58.62	24.84	21.00	26.63	24.24	46.90	76.35	12.32	202.25	278.56
*1.6*	Steinert	75.34	24.28	20.20	28.46	25.60	46.68	76.75	12.38	220.51	297.29
	Control	77.35	23.37	19.20	26.00	23.81	45.58	76.17	12.14	215.23	291.42
*1.7*	Steinert	91.63	23.85	20.54	26.69	24.69	44.49	76.23	11.86	231.68	307.91
	Control	95.98	21.88	17.76	25.15	22.76	44.03	75.85	11.87	226.55	302.42
*1.8*	Steinert	86.50	23.13	20.55	27.69	24.44	44.53	76.39	12.00	226.67	303.08
	Control	91.42	21.40	16.86	25.13	22.66	44.97	75.85	11.92	222.38	298.28
*2.1*	Steinert	27.88	25.23	20.78	26.40	23.98	49.55	76.05	11.38	173.95	250.08
	Control	24.15	23.77	19.59	24.87	22.82	47.97	75.98	11.78	163.14	239.11
*2.2*	Steinert	32.40	27.88	23.20	28.06	24.88	51.02	77.00	11.97	187.35	264.35
	Control	28.14	26.87	22.17	26.87	23.68	49.15	76.20	11.98	176.96	253.15
*2.3*	Steinert	37.05	30.91	25.52	31.08	26.72	52.91	77.69	12.45	204.15	281.82
	Control	32.85	30.41	24.98	29.87	25.39	50.62	76.62	12.33	194.08	270.69
*2.4*	Steinert	41.03	34.86	28.82	33.52	28.45	54.80	78.26	12.85	221.43	299.78
	Control	37.21	33.64	28.11	32.02	27.42	51.90	76.88	12.54	210.40	287.35
*2.5*	Steinert	46.25	35.31	29.15	34.20	29.17	55.08	78.74	13.22	229.14	307.85
	Control	42.14	34.58	28.98	32.37	28.03	52.38	77.17	12.74	218.60	295.66
*2.6*	Steinert	55.18	32.72	26.58	33.32	27.85	53.68	78.62	13.20	229.48	308.05
	Control	51.95	31.68	25.75	31.35	27.17	51.82	77.06	12.62	219.58	296.64
*2.7*	Steinert	77.58	28.05	22.60	30.20	26.88	50.08	78.08	12.80	235.43	313.51
	Control	77.68	26.60	21.10	28.95	25.26	48.47	76.73	12.32	228.09	304.84
*2.8*	Steinert	109.52	23.94	19.78	27.30	24.70	45.06	77.28	12.25	250.30	327.53
	Control	113.97	22.74	18.03	25.84	23.29	44.19	76.53	12.07	248.12	324.66
*3.1*	Steinert	22.89	29.86	25.30	29.48	25.55	50.98	76.95	11.44	184.13	261.02
	Control	19.95	27.61	23.57	27.48	24.12	50.25	76.14	11.55	173.02	249.18
*3.2*	Steinert	25.48	36.35	29.97	32.95	27.80	54.57	78.22	12.14	207.12	285.32
	Control	22.69	34.29	28.52	31.48	26.03	53.16	76.68	12.15	196.19	272.95
*3.3*	Steinert	28.66	45.37	35.60	37.69	30.68	60.37	79.20	12.85	238.48	317.72
	Control	25.88	43.07	34.79	37.01	29.08	58.06	77.49	12.75	227.95	305.46
*3.4*	Steinert	32.78	53.43	40.95	41.82	33.55	63.89	80.17	13.60	266.31	346.48
	Control	29.62	51.05	40.32	40.52	33.27	60.38	78.12	13.27	255.06	333.22
*3.5*	Steinert	35.37	53.74	42.08	42.43	35.72	64.09	80.68	14.11	273.35	354.00
	Control	32.81	52.11	40.99	41.12	35.23	60.23	78.40	13.45	262.38	340.83
*3.6*	Steinert	43.37	49.05	38.03	40.29	34.02	61.11	80.42	13.88	265.80	346.15
	Control	39.88	47.23	36.62	38.73	32.01	59.17	78.25	13.29	253.48	331.69
*3.7*	Steinert	57.83	38.26	30.05	35.72	30.62	56.20	79.82	13.48	248.52	328.29
	Control	53.82	37.10	28.91	33.26	28.58	54.50	77.72	12.87	236.18	313.95
*3.8*	Steinert	91.73	29.09	23.25	31.19	27.48	48.92	78.72	12.77	251.84	330.45
	Control	91.47	27.87	22.19	29.11	25.74	47.92	77.08	12.25	244.33	321.43
*4.1*	Steinert	18.78	31.70	29.13	30.80	27.39	52.05	77.83	11.56	189.73	267.69
	Control	17.22	29.06	28.15	29.59	25.45	51.66	76.65	11.52	181.02	257.71
*4.2*	Steinert	18.74	41.26	35.89	35.69	29.92	58.18	79.28	12.35	219.63	298.86
	Control	17.67	38.95	34.41	34.99	28.07	57.23	77.46	12.28	211.39	288.84
*4.3*	Steinert	19.05	54.17	43.37	40.02	33.71	66.58	81.00	13.55	257.05	337.95
	Control	17.98	51.65	42.22	39.82	31.27	66.12	78.84	13.12	249.02	327.83
*4.4*	Steinert	18.43	45.55	39.58	37.46	34.77	76.42	84.94	14.77	252.06	337.11
	Control	16.95	41.86	36.84	35.26	34.98	73.87	82.98	14.80	239.71	322.72
*4.5*	Steinert	15.85	34.28	32.43	32.23	34.14	85.71	87.91	15.82	234.43	322.22
	Control	15.22	34.16	32.33	32.17	35.71	79.22	85.14	15.77	228.57	313.75
*4.6*	Steinert	23.06	56.89	45.71	44.14	37.88	70.91	83.78	14.95	278.51	362.43
	Control	21.59	55.22	44.12	42.67	35.30	69.38	80.97	14.36	268.30	349.32
*4.7*	Steinert	38.23	49.62	38.26	40.34	35.25	61.37	81.35	13.92	263.05	344.40
	Control	34.95	46.34	36.18	37.96	31.68	60.30	78.98	13.20	247.42	326.35
*4.8*	Steinert	62.92	34.37	27.22	34.45	31.55	50.17	79.23	12.32	240.57	319.86
	Control	59.16	32.88	26.22	32.08	28.64	49.38	78.28	12.70	228.40	306.71
*5.1*	Steinert	18.80	26.84	30.77	29.77	27.53	52.86	78.36	11.81	186.56	264.8
	Control	17.77	23.73	30.01	28.52	25.29	52.49	77.11	11.75	177.79	254.92
*5.2*	Steinert	17.71	36.23	37.35	34.55	29.74	60.20	79.94	12.65	215.68	295.62
	Control	17.71	33.58	35.46	33.52	27.46	59.54	78.03	12.38	207.32	285.30
*5.3*	Steinert	15.23	51.14	44.31	39.31	32.58	69.78	81.52	13.57	252.35	333.91
	Control	15.16	48.53	42.58	38.05	29.67	70.27	79.63	13.49	244.27	323.88
*5.4*	Steinert	16.92	43.51	38.62	36.57	34.55	77.48	85.28	14.95	247.68	332.95
	Control	16.18	39.44	35.92	33.18	29.55	81.27	83.59	15.15	235.34	318.95
*5.5*	Steinert	15.69	35.83	32.95	33.20	36.18	81.34	87.38	15.86	234.85	322.37
	Control	15.33	35.78	33.16	32.1	31.77	82.61	85.25	15.95	230.26	315.57
*5.6*	Steinert	24.09	57.18	45.55	44.31	37.23	70.82	84.20	15.15	279.09	363.34
	Control	23.74	55.02	43.49	42.59	34.37	70.11	81.03	14.49	268.97	349.95
*5.7*	Steinert	40.60	48.62	37.45	40.23	33.72	63.20	81.51	13.86	263.89	345.28
	Control	38.86	45.02	35.02	38.10	30.82	61.42	79.09	13.34	249.01	328.12
*5.8*	Steinert	64.97	33.15	26.75	33.71	29.85	51.98	79.43	12.52	240.49	319.95
	Control	63.42	31.43	24.63	31.38	27.39	51.47	78.18	12.48	229.50	307.64
*6.1*	Steinert	17.98	27.66	27.83	28.80	26.28	52.97	78.39	11.84	181.34	259.73
	Control	16.11	24.52	26.14	27.93	24.23	52.41	77.15	11.85	171.38	248.54
*6.2*	Steinert	18.55	36.55	31.63	33.17	27.37	57.89	79.29	12.35	205.18	284.46
	Control	17.26	32.66	30.12	32.05	25.32	57.25	77.61	12.18	194.70	272.30
*6.3*	Steinert	21.12	46.55	36.26	37.82	29.49	64.34	80.28	13.11	235.62	315.89
	Control	20.12	43.22	34.72	36.32	27.62	64.07	78.47	12.89	226.15	304.58
*6.4*	Steinert	27.43	54.34	40.72	40.78	31.74	69.66	81.38	13.97	264.63	346.05
	Control	25.68	51.39	39.66	39.52	28.92	70.12	79.42	13.66	255.26	334.62
*6.5*	Steinert	34.45	54.49	42.32	41.80	33.54	69.91	82.35	14.83	276.43	358.75
	Control	31.96	52.12	41.08	40.19	30.12	70.36	80.06	14.33	265.94	345.94
*6.6*	Steinert	42.62	48.43	37.72	40.11	31.85	67.00	81.88	14.43	267.66	349.54
	Control	40.16	45.79	35.73	38.47	29.11	66.22	79.49	13.77	255.52	335.08
*6.7*	Steinert	54.95	38.58	29.88	35.68	29.25	62.29	80.92	13.69	250.46	331.49
	Control	52.72	36.23	28.42	33.76	26.72	60.59	78.78	13.06	238.50	317.20
*6.8*	Steinert	78.57	30.03	23.94	31.78	27.00	56.14	79.52	12.65	247.51	327.09
	Control	78.35	27.95	22.25	29.87	24.75	55.92	77.96	12.32	239.02	316.99
*7.1*	Steinert	20.88	25.75	22.09	27.16	24.56	52.69	78.08	11.66	173.11	251.27
	Control	17.12	23.68	20.39	25.78	23.22	52.04	77.32	11.67	162.34	239.62
*7.2*	Steinert	23.51	30.23	24.92	29.28	25.51	55.69	78.74	12.05	189.12	267.80
	Control	19.67	27.98	23.52	27.88	23.88	55.25	77.78	12.12	178.11	255.88
*7.3*	Steinert	27.77	34.45	27.80	31.74	26.52	59.37	79.69	12.74	207.66	287.38
	Control	24.38	32.39	26.68	30.32	24.82	58.98	78.35	12.63	197.59	275.90
*7.4*	Steinert	33.74	37.98	31.32	33.40	27.71	63.42	80.46	13.4	227.42	308.02
	Control	30.42	36.30	29.85	32.35	25.55	63.16	78.78	13.05	217.54	296.35
*7.5*	Steinert	42.11	38.77	32.06	34.03	27.92	64.69	81.14	13.89	239.57	320.66
	Control	38.71	36.87	30.70	32.58	25.75	64.30	79.15	13.29	228.78	307.95
*7.6*	Steinert	50.60	35.05	28.86	33.91	27.34	62.32	81.05	13.89	238.29	319.28
	Control	48.28	32.81	26.68	31.34	25.12	61.95	78.95	13.18	226.19	305.16
*7.7*	Steinert	62.85	29.65	24.69	32.91	26.62	59.20	80.32	13.26	235.86	316.18
	Control	62.75	27.75	22.29	29.37	24.24	58.99	78.65	12.84	225.35	303.98
*7.8*	Steinert	86.80	26.11	22.02	31.02	25.43	54.97	79.45	12.65	246.23	325.77
	Control	92.82	23.62	18.87	27.55	23.31	54.66	78.25	12.42	240.83	319.09
*8.1*	Steinert	23.61	22.69	18.98	25.92	24.47	51.98	77.42	11.41	167.58	245.08
	Control	20.91	21.54	17.84	23.97	22.90	51.77	77.41	11.87	158.95	236.28
*8.2*	Steinert	28.15	24.45	20.08	26.98	24.42	54.12	78.09	11.74	178.05	256.15
	Control	24.16	23.50	19.51	25.28	22.93	53.92	77.55	12.08	169.29	246.78
*8.3*	Steinert	33.43	26.60	21.74	28.11	24.34	55.92	78.78	12.40	190.08	268.89
	Control	29.38	25.36	21.01	26.15	23.29	56.00	77.68	12.24	181.35	259.08
*8.4*	Steinert	39.05	28.63	24.15	29.11	25.02	57.83	79.37	12.77	203.69	283.05
	Control	35.52	27.10	22.95	27.04	23.44	58.08	77.95	12.36	194.14	272.05
*8.5*	Steinert	46.12	29.92	25.48	29.91	25.02	58.42	79.83	13.09	214.92	294.77
	Control	43.78	27.92	23.77	27.67	23.40	58.61	78.15	12.59	205.17	283.41
*8.6*	Steinert	56.26	28.71	24.12	29.45	24.97	57.58	79.80	13.03	220.94	300.77
	Control	56.60	25.85	21.58	27.13	23.12	57.70	78.16	12.52	212.05	290.21
*8.7*	Steinert	72.02	26.22	21.66	28.28	24.75	55.95	79.40	12.74	228.80	308.23
	Control	76.38	23.22	18.68	26.07	22.85	55.35	77.96	12.39	222.69	300.60
*8.8*	Steinert	88.94	24.85	20.25	27.63	23.91	52.55	78.83	12.28	238.18	317.08
	Control	93.08	21.73	17.29	26.05	22.27	53.27	77.81	12.20	233.54	311.36
*Nsite*	pRNFL	Global		PMB	NT_Ratio	NS	N	NI	TI	T	TS
	Steinert	104.48		58.87	1.16	112.48	84.69	111.62	145.74	75.52	145.43
	Control	104.53		53.64	1.23	111.61	85.47	123.10	147.79	71.44	141.21
	p-value	1.0		0.000	1.0	1.0	1.0	1.0	1.0	0.028	1.0

RNFL, retinal nerve fiber layer; GCL, ganglion cell layer; IPL, inner plexiform layer; OPL, outer plexiform layer; ONL, outer nuclear layer; Out_Ret, outer retina; RPE, retina pigment epithelium; Inner_Ret, inner retina; pRNFL, peripapillary retinal nerve fiber layer; G, general; PMB, papillomacular bundle; NT, nasal/temporal; NS, nasal superior; N, nasal; NI, nasal inferior; TI, temporal inferior; TS, temporal superior.

Yellow shading indicates lower retinal thickness statistically significant differences (p < 0.05) in the Steinert disease group than in control group. Green shading indicates greater retinal thickness statistically significant differences (p < 0.05) in the Steinert disease group than in the control group.

**Figure 3** shows graphically the statistically significant differences in the retinal layers by cells and optic nerve sectors in Steinert disease group vs control group. OCT-based Ppole analysis showed in both Steinert and control groups a retinal thickness within the normal range (16) (in green). A mild significant increase in thickness was observed in most layers in patients with Steinert disease. And a few isolated cells (in yellow, blue, red and purple) showed a decrease in thickness in Steinert disease, specifically in the RNFL layers (cells 1.6, 1.7, 1.8, 2.7, 2.8) and the outer layers, OPL (cell 4.5), ONL (cell 5.3), and RPE (cells 4.8 and 6.1). OCT-based Nsite analysis showed significant thickening of the peripapillary RNFL in the temporal (p<0.028) area of the optic nerve and in the area of the papillomacular bundle (p<0.001) (**Table 1**). This can be seen visually in **Figure 4** where the pvalue values are represented with a color scale in each of the retinal layers (**Figure 4**).

**Figure 3:**
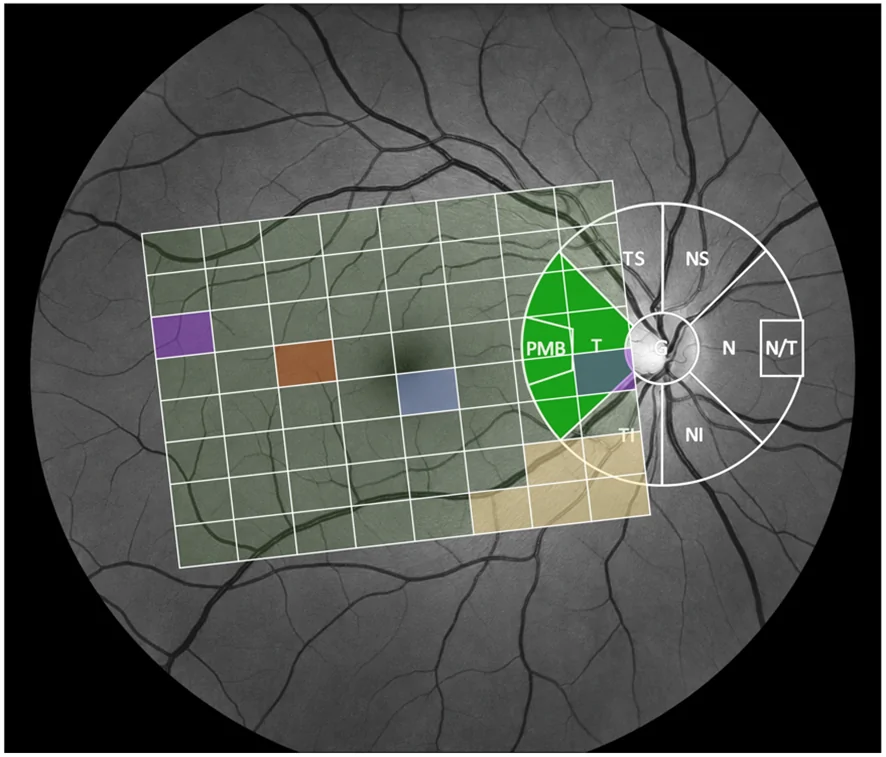
Graphical representation of the PPole and Nsite Spectralis scanning grids superimposed on a fundus image. Green areas indicate regions with significantly increased retinal thickness in patients with Steinert disease compared with healthy controls. Regions showing significantly reduced thickness in the Steinert group compared with controls are color-coded according to the affected retinal layer, yellow, retinal nerve fiber layer (RNFL); purple, retinal pigment epithelium (RPE); blue, outer plexiform layer (OPL); and red, outer nuclear layer (ONL).

**Figure 4:**
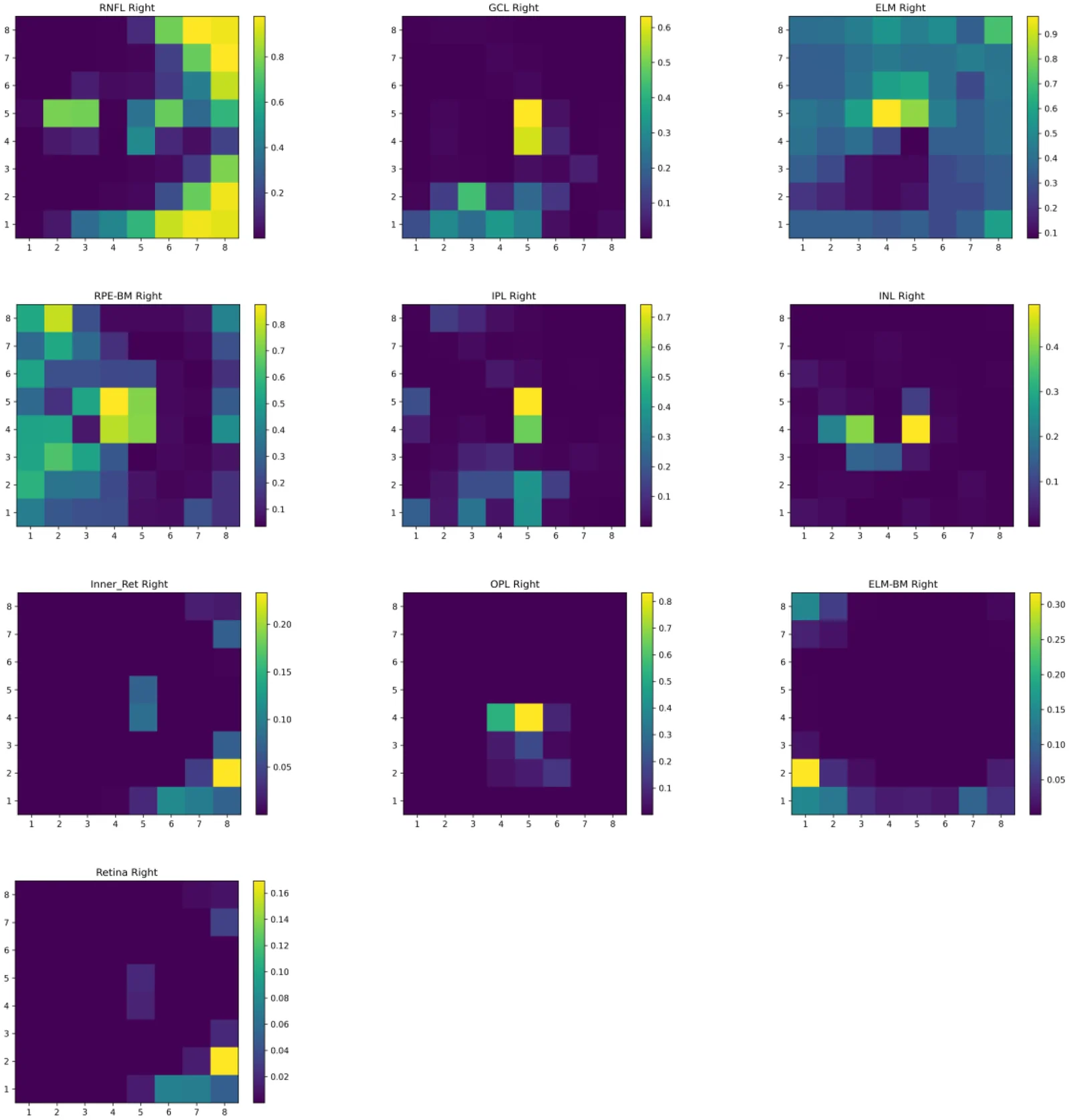
Significance data produced by the Mann–Whitney U test for each cell of the posterior pole protocol grid in right eye. P-values were adjusted using the Benjamini-Hochberg false discovery rate (FDR) procedure (q = 0.05). Cold colors indicate a lower p-value and warm colors indicate a higher p-value.

## Discussion

The purpose of this study was to evaluate the different retinal layers of the posterior pole and peripapillary areas to identify retinal structural changes associated with Steinert disease.

Most studies regarding Steinert disease focused on the eye refer or eyelid ptosis and ocular motility changes due to muscle alteration. Steinert disease or myotonic dystrophy type 1 It is a disease traditionally considered to be muscular, but today it is recognized as a multisystem disorder that also affects the central nervous system (17). In this sense, recent studies have shown involvement of other parts of the eye, such as cataracts (18) or the cornea, in connection with Fuchs’ endothelial dystrophy (19), as well as isolated cases of retinal involvement. In a study analyzing electroretinograms taken of these patients, a decrease in the *b*-wave was observed under both scotopic and photopic conditions. This wave corresponds to bipolar cells and helps explain the functional night-vision and dark-adaptation deficits frequently observed in patients with myotonic dystrophy (20).

Furthermore, it has been proposed that Steinert disease acts as a form of premature aging, in which the genetic expansion of the CTG triplet leads to the accumulation of toxic RNA that sequesters regulatory proteins, causing widespread failures in the alternative splicing of essential genes and causing cells to enter a state of cellular senescence. This disrupts the neuronal and synaptic function of glial cells as astrocyte and oligodendrocytes (17) and has been linked to the accumulation of dysfunctional proteins such as tau and amyloid, which are present in Parkinson’s and Alzheimer’s diseases.

The retina is considered a visible extension of the brain. To date very little research focuses on the retina in these patients. Indeed, there is very little literature analyzing OCT-measured retinal layer thicknesses in patients with Steinert disease. Only one study using OCT reported diffuse subretinal hyperreflective material between the photoreceptor layer and the RPE, which could explain this increase in thickness in the outermost layers (21).

Our study provides the largest sample size in this population to date and compares it against a group of control subjects of the same age. Furthermore, the exclusion of concomitant retinal pathologies, such as epiretinal membranes and macular edema, represents an important distinction from previous studies and allows retinal thickness changes associated with Steinert disease to be assessed while minimizing potential confounding factors. The findings of our study reveal an interesting pattern of neuroretinal changes. The generalized mild thickening of the retinal layers observed in patients with Steinert disease in our study has also been reported in other neurological disorders, such as Parkinson’s disease, and may be related to the accumulation of tau-like misfolded proteins (22). However, histological studies would be needed to corroborate this hypothesis. Significant thickening of the peripapillary RNFL was also found in the temporal area of the optic nerve and in the papillomacular bundle, which comprises the parvocellular retinal ganglion cell axons connecting the macula with the optic nerve. This finding is of particular interest because RNFL thinning, including preferential involvement of the temporal RNFL and papillomacular bundle, has been described in several neurodegenerative disorders, such as in multiple sclerosis or Parkinson’s disease (23, 24). However, an increase in thickness due to RNFL hypertrophy has been reported in other neurological disorders, such as Charlevoix-Saguenay Ataxia. Our findings show thickening in this region of the optic nerve, suggesting that the structural changes observed in Steinert disease may show a differential pattern from the previous described in other neurodegenerative conditions.

One of the limitations of this study is that, although the sample size is large, a larger number of subjects would make the results more conclusive. Ideally, the average of both eyes would be used to provide more information. We ruled out using both eyes in this population because of the possibility of OCT value interdependence between right and left eyes since both eyes correspond to the same patient and are subject to the same genetic and environmental conditioning factors.

Another limitation is that, since we only used the Spectralis device, our results are only extrapolatable to a population evaluated with this same OCT device and cannot be extrapolated to populations evaluated using other similar devices produced by other manufacturers.

A further possible limitation is that cataracts are quite common in Steinert disease. To minimize this limitation and the potential influence of cataracts on the retinal thickness values, we only included patients with an incipient degree of phacosclerosis (corresponding to grade one on the LOCS scale) and patients who had undergone cataract surgery at least 6 months previously to avoid the possible presence of retinal inflammation secondary to surgery.

A final limitation is that our study did not include follow-up; future studies should evaluate the change in OCT values over the course of the disease, and analyze whether OCT can also be a useful tool in following up and monitoring the effect of the treatment on these subjects.

Overall, our findings demonstrate significant differences in retinal layer thickness between patients with Steinert disease and controls, supporting the presence of retinal structural alterations associated with the disease. These findings suggest that OCT may provide potentially relevant structural markers of retinal involvement in Steinert disease. However, our cross-sectional study was not designed to assess diagnostic accuracy, and further studies are needed to determine whether these OCT findings may have clinical utility as biomarkers or contribute to disease characterization. Future studies should also investigate whether the retinal structural changes observed in Steinert disease are associated with relevant clinical and genetic characteristics, including disease severity, neurological status, and visual function. Given the limitations in accurately determining disease onset and duration in this population, particularly in patients with cognitive impairment, longitudinal studies incorporating objective clinical and genetic measures may help clarify the clinical significance of these OCT findings and further evaluate their potential as structural markers of disease.

## Data Availability

The original contributions presented in the study are included in the article/supplementary material. Further inquiries can be directed to the corresponding author/s.

## References

[1] Burian HM Burns CA . Ocular changes in myotonic dystrophy. Am J Ophthalmol. (1967) 63:22–34. doi: 10.1016/0002-9394(67)90577-6 6017679

[2] Gutiérrez Gutiérrez G Díaz-Manera J Almendrote M Azriel S Eulalio Bárcena J Cabezudo García P . Clinical guide for the diagnosis and follow-up of myotonic dystrophy type 1, MD1 or Steinert’s disease. Neurologia. (2020) 35:185–206. doi: 10.1016/j.nrl.2019.01.001 31003788

[3] Theadom A Rodrigues M Roxburgh R Balalla S Higgins C Bhattacharjee R . Prevalence of muscular dystrophies: a systematic literature review. Neuroepidemiology. (2014) 43:259–68. doi: 10.1159/000369343 25532075

[4] Anastasopoulos D Kimmig H Mergner T Psilas K . Abnormalities of ocular motility in myotonic dystrophy. Brain. (1996) 119:1923–32. doi: 10.1093/brain/119.6.1923 9009998

[5] Clauser L Tieghi R Galie M . Palpebral ptosis: clinical classification, differential diagnosis, and surgical guidelines: an overview. J Craniofac Surg. (2006) 17:246–54. doi: 10.1097/00001665-200603000-00008 16633170

[6] García-Cruz I Muñoz-Negrete FJ Benito-Pascual B Arriola-Villalobos P Pérez-Bañón MD Alió JL . Ocular findings in a Spanish cohort of myotonic dystrophy type 1. Graefes Arch Clin Exp Ophthalmol. (2023) 261:1109–14. doi: 10.1007/s00417-022-05875-4 36331602

[7] Kidd A Turnpenny P Kelly K Clark C Church W Hutchinson C . Ascertainment of myotonic dystrophy through cataract by selective screening. J Med Genet. (1995) 32(7):519–23. doi: 10.1136/jmg.32.7.519 7562963 PMC1050543

[8] Kimizuka Y Kiyosawa M Tamai N Takase S . Retinal changes in myotonic dystrophy. Clinical and followup evaluation. Retina. (1993) 13(2):129–35. 8337494

[9] Kim US Kim JS Hwang JM . A case of myotonic dystrophy with pigmentary retinal changes. Korean J Ophthalmol. (2009) 23:121–3. doi: 10.3341/kjo.2009.23.2.121 19568364 PMC2694290

[10] Schuman JS Pedut-Kloizman T Hertzmark E Hee MR Wilkins JR Coker JG . Reproducibility of nerve fiber layer thickness measurements using optical coherence tomography. Ophthalmology. (1996) 103:1889–98. doi: 10.1016/s0161-6420(96)30410-7 8942887 PMC1939724

[11] Vasilijevic J Peric S Basta I Kovacevic I Maric G Avram N . Retinal vascular abnormalities in myotonic dystrophy assessed by optical coherence tomography angiography - Cross-sectional study. Eur J Ophthalmol. (2025) 35:262–8. doi: 10.1177/11206721241247424 38644803

[12] Abed E D'Amico G Rossi S Perna A Bianchi MLE Silvestri G . Spectral domain optical coherence tomography findings in myotonic dystrophy. Neuromuscul Disord. (2020) 30:144–50. doi: 10.1016/j.nmd.2019.11.012 32005494

[13] Chylack LT Jr Wolfe JK Singer DM Leske MC Bullimore MA Bailey IL . The lens opacities classification system III. The longitudinal study of cataract study group. Arch Ophthalmol. 111(6):831–6. doi: 10.1001/archopht.1993.01090060119035 8512486

[14] Balasubramanian M Bowd C Vizzeri G Weinreb RN Zangwill LM . Effect of image quality on tissue thickness measurements obtained with spectral domain-optical coherence tomography. Opt Express. (2009) 17:4019–36. doi: 10.1364/oe.17.004019 19259243 PMC2848174

[15] Kuntawala DH Vitorino R Cruz AC Martins F Rebelo S . Multisystem symptoms in myotonic dystrophy type 1: A management and therapeutic perspective. Int J Mol Sci. (2025) 26:5350. doi: 10.3390/ijms26115350 40508159 PMC12155408

[16] Palazon-Cabanes A Palazon-Cabanes B Rubio-Velazquez E Lopez-Bernal MD Garcia-Medina JJ Villegas-Perez MP . Normative database for all retinal layer thicknesses using SD-OCT posterior pole algorithm and the effects of age, gender and axial lenght. J Clin Med. (2020) 9:3317. doi: 10.3390/jcm9103317 33076558 PMC7602827

[17] Liu J Guo Z-N Yan X-L Yang Y Huang S . Brain pathogenesis and potential therapeutic strategies in myotonic dystrophy type 1. Front Aging Neurosci. (2021) 13:755392. doi: 10.3389/fnagi.2021.755392 34867280 PMC8634727

[18] Scutifero M Lanza M Petillo R De Bernardo M Passamano L Rosa N . Gender effect on onset, prevalence and surgical treatment of cataract in patients with Myotonic Dystrophy type 1. Acta Myol. (2022) 41(3):105–10. doi: 10.36185/2532-1900-N75 36349183 PMC9628803

[19] Landi E Wansink DG LaPointe V van Bokhoven H Davidson AE Dickman M . Pathological mechanism in Fuchs endothelial corneal dystrophy and myotonic dystrophy type 1: more than meets the eye. Prog Retin Eye Res. (2026) 110:101418. doi: 10.1016/j.preteyeres.2025.101418 41285302

[20] Stanescu B Michiels J . Temporal aspects of electroretinography in patients with myotonic dystrophy. Am J Ophthalmol. (1975) 80:224–6. doi: 10.1016/0002-9394(75)90136-1 1155561

[21] Esteves F Dolz-Marco R Hernández-Martínez P Díaz-Llopis M Gallego-Pinazo R . Pattern dystrophy of the macula in a case of Steinert disease. Case Rep Ophthalmol. (2013) 4:129–33. doi: 10.1159/000355385 24163680 PMC3806677

[22] Rossi S Silvestri G . Fluid biomarkers of central nervous system (CNS) involvement in myotonic dystrophy type 1 (DM1). Int J Mol Sci. (2023) 24:2204. doi: 10.3390/ijms24032204 36768526 PMC9917343

[23] Yu JG Feng YF Xiang Y Huang JH Savini G Parisi V . Retinal nerve fiber layer thickness changes in Parkinson disease: a meta-analysis. PLoS One. (2014) 9:e85718. doi: 10.1371/journal.pone.0085718 24465663 PMC3897496

[24] Garcia-Martin E Pueyo V Martin J Almarcegui C Ara JR Dolz I . Progressive changes in the retinal nerve fiber layer in patients with multiple sclerosis. Eur J Ophthalmol. (2010) 20:167–73. doi: 10.1177/112067211002000123 19882541

